# Moulding

**Published:** 1850-01

**Authors:** E. S. Holmes


### MOULDING.

DR. TAYLOR,

I have for about two years past, made use of an article for moulding, which I consider far superior to anything we find mentioned in "the books," or by our instructors, for that purpose. It is common Spanish whiting, used dry, or containing no more moisture than it absorbs from the humidity of the atmosphere.

The principal advantage gained by the use of whiting is the *beauty and perfection* of the cast taken from it, which is a desideratum with many operators, particularly in atmospheric pressure sets.

The whiting should be made free from lumps, (it requires no other preparation,) and used the same as sand.

If you consider the above information of any importance to the Dental profession, if it will add one item to the many facilities which we possess more than our fathers did; or if it will make any advancement toward that professional perfection at which every Dentist ought to aim, you are at liberty to make such use of it as will best subserve these important interests.

E. S. HOLMES.

